# Exploring the Impact of Linguistic Signals Transmission on Patients’ Health Consultation Choice: Web Mining of Online Reviews

**DOI:** 10.3390/ijerph18199969

**Published:** 2021-09-22

**Authors:** Adnan Muhammad Shah, Mudassar Ali, Abdul Qayyum, Abida Begum, Heesup Han, Antonio Ariza-Montes, Luis Araya-Castillo

**Affiliations:** 1 Department of Management Sciences, Shaheed Zulfikar Ali Bhutto Institute of Science and Technology, Islamabad 44320, Pakistan; dr.adnan.shah@szabist-isb.edu.pk; 2 Charles E. Schmidt College of Science, Florida Atlantic University, Boca Raton, FL 33431-0991, USA; 3 School of Management, Harbin Institute of Technology, Harbin 150001, China; mudassarsalamat@gmail.com; 4 Faculty of Management Science, Riphah International University, Rawalpindi 46000, Pakistan; abdul.qayyum@riphah.edu.pk; 5 School of Marxism, Northeast Forestry University, Harbin 150040, China; abidakhg@gmail.com; 6 College of Hospitality and Tourism Management, Sejong University, 98 Gunja-Dong, Gwanjin-Gu, Seoul 143-747, Korea; 7 Social Matters Research Group, Universidad Loyola Andalucía, C/Escritor Castilla Aguayo, 4, 14004 Córdoba, Spain; ariza@uloyola.es; 8 Facultad de Economía y Negocios, Universidad Andrés Bello, Santiago de Chile 7591538, Chile; luis.araya@unab.cl

**Keywords:** online review helpfulness, signaling theory, sentiment analysis, physician rating websites, consumer decision-making, COVID-19

## Abstract

Background: Patients face difficulties identifying appropriate physicians owing to the sizeable quantity and uneven quality of information in physician rating websites. Therefore, an increasing dependence of consumers on online platforms as a source of information for decision-making has given rise to the need for further research into the quality of information in the form of online physician reviews (OPRs). Methods: Drawing on the signaling theory, this study develops a theoretical model to examine how linguistic signals (affective signals and informative signals) in physician rating websites affect consumers’ decision making. The hypotheses are tested using 5521 physicians’ six-month data drawn from two leading health rating platforms in the U.S (i.e., Healthgrades.com and Vitals.com) during the COVID-19 pandemic. A sentic computing-based sentiment analysis framework is used to implicitly analyze patients’ opinions regarding their treatment choice. Results: The results indicate that negative sentiment, review readability, review depth, review spelling, and information helpfulness play a significant role in inducing patients’ decision-making. The influence of negative sentiment, review depth on patients’ treatment choice was indirectly mediated by information helpfulness. Conclusions: This paper is a first step toward the understanding of the linguistic characteristics of information relating to the patient experience, particularly the emerging field of online health behavior and signaling theory. It is also the first effort to our knowledge that employs sentic computing-based sentiment analysis in this context and provides implications for practice.

## 1. Introduction

Recent developments in digitalization have opened up doors to the health sector. An increasing number of people are turning to the Internet to collect information related to their healthcare needs [1]. One of the popular sources for seeking and sharing health-rated information is the physician rating websites (PRWs). Patients search for information regarding past patients’ experience with physicians and their working practices from these rating sites [2]. PRWs provide patients with a unique platform to post their quality of service encounters and write reviews of healthcare providers. From a physician’s perspective, PRWs are valuable because the expectations of patients about the level of care rendered by the physician are made publicly accessible. Hence, PRWs have become a powerful source of information in patients’ choice for physician selection, as consumers used to rely on online reviews to make purchase decisions [3]. Recently, increasing the utilization of PRWs is becoming popular among healthcare consumers. Health professionals need to learn new ways in which different information aspects of PRWs affect patients’ decision-making process.

Recent marketing research has reported that online reviews influence not only customer choice [4] but product sales as well [5]. Researchers have carried out several empirical studies in different domains, such as tourism [6], hotel booking [7], and e-commerce [5]. Online reviews have also gained interest from researchers in the healthcare field. A variety of investigations were performed using different PRWs in different countries, such as the average rating score of a physician, review volume [8,9,10], and qualitative analysis of OPRs to mine patients’ interests [11]. In addition to these studies, several other studies have examined the impact of physician star ratings and textual feedback on patients’ choice [10,12,13,14]. Similarly, online physician reviews (OPRs) posted by patients on PRWs provide recommendations for a physician in enhancing the quality of care. Despite there being few academic studies about how to apply online information to identify good doctors, it is complicated to determine how best to assess their relevance. Moreover, information quality measurement is not yet complete, and its effects on patients’ consultation choice are largely ignored. Based on previous studies, healthcare practitioners still have a shortage of comprehensive frameworks to assist them in developing IT solutions to help find excellent doctors. Therefore, it is critically important for healthcare providers to evaluate whether and to what degree the various service characteristics lead to overall physician satisfaction among patients [15].

In addition, scant research has been performed on how OPRs affect patients’ health consultation decision-making process. Although, academics analyzed quantitative patient ratings posted on PRWs regarding patient healthcare choice [12]; however, a patient’s real behavior and emotional response could not be captured from quantitative ratings, as a result of valuable information loss. Furthermore, feedback comments are more valued than numerical ratings in reviews. Hence, it is of particular interest to the researchers that patients use online linguistic signals (unstructured comments) to evaluate the information diagnosticity in choosing a good doctor [16]. As an online signal, linguistic signaling is an appealing and multifaceted subject, particularly in an online environment where there are often considerable asymmetries to information. Patients post OPRs on PRWs regarding a physician’s healthcare quality, whereas the physician invokes a response in the form of an answer to the review. This communication between doctors and patients can be a major challenge that leads to weak ties, social and geographical distance that characterizes PRWs. However, in the perspective of PRWs, there is no theoretical model available in our knowledge that considers signaling in relation to information patterns. Since linguistic patterns have not been understudied in previous e-health literature and thus require further investigation, these are likely to play an important role in evaluating the physicians’ performance, in turn, lead to positive patients’ behavior.

Given the motivation for our research and the nascent nature of information technology in the healthcare domain, we propose a method for identifying good physicians using big data analytics-based sentic computing framework by combining different linguistic signals to recommend suitable and high-quality physicians in PRWs. The proposed approach is based on available physician data in a big data context and connects patients to high-quality physicians in order to improve care services. Identifying the right doctor can give patients peace of mind, helping them feel better about the choice they have made [17]. Involving patients in the organization and process is known to strengthen the doctor-patient relationship, leading to fewer tensions and incidents in hospitals, resulting in fewer lawsuits. This research, therefore, aims at contributing to the literature by drawing a theoretical framework on the basis of signaling theory. The current study utilizes linguistic signaling in the form of affective features (negative sentiment) and informative features (readability, depth, spelling, and review helpfulness) to investigate their impacts on patients’ treatment choice. As recommendations play an important role in patients’ decision-making, we also investigated different linguistic antecedents of information helpfulness (IH) in online healthcare services. Next, the mediation effect of IH onto the relationships between linguistic signals and patients’ treatment choice was also investigated. After investigating the differential effects on the outcome variable, we analyze patients’ emotions implicitly from OPRs using a sentic computing framework [18]. The proposed model is tested using unique datasets from two famous PRWs in the U.S (Healthgrades and Vitals), which covers the COVID-19 outbreak period from December 2019–June 2020. This research also provides guidance for platform developers, management of PRWs, and physicians to include essential information components on PRWs, which could improve patients’ behavior toward a physician.

## 2. Theoretical Background and Research Hypothesis

### 2.1. Linguistic Patterns

PRWs contain plenty of linguistic signals, which are a precious resource for people who are seeking health information and support [19]. Previous studies regarding the effects of *information quality* as a linguistic signal refer to the persuasive strength of the message, which is commonly measured in terms of its relevance, timeliness, accuracy, and comprehensiveness [20]. Reviews posted by different users are always different in length, accuracy, comprehensiveness, tone, and even logic [21]. In an online environment, users perceived the information regarding a particular activity in which they were engaged, fitted in their expectations and requirements [22].

Extent literature employed “argument quality or information quality” to measure its effect on the users’ behavior [21,23,24]. Since the last few years, researchers have been struggling to better understand the impact of online rating sites on various aspects of people’s choice behaviors [4,23,25]. Following this line of research, information evaluation is considered an important antecedent of the patients’ healthcare decisions. From the perspective of traditional communication theories, patients evaluate information from different perspectives before making their health consultation decisions [10]. Online reviews integrate information from various sources providing online word-of-mouth (WOM) to healthcare consumers who lack WOM [13]. Extant studies on the patients’ choice of physician outlined that information gathered from peers or other patients are always amongst the top influencing factors [9,26].

In line with this research, several other studies have also stated the information quality as a predictor of users’ behavior in the healthcare domain [27,28]. The healthcare field is categorized by high environmental and demand uncertainty, and healthcare consumers and providers are more likely to look for appropriate and credible information [29]. Therefore, efficient information management contributes to the customer benefits in healthcare [30]. These arguments are further supported by Wu [21], who stated that information quality is highly a critical factor that influences the patients’ online behavior. Moreover, Yoon [31] stated that patients tend to adopt that information, which is more factual, relevant, and useful. Hence, quality information about a doctor enables patients to obtain further information on healthcare providers and hospitals. For instance, a study by Lu and Zhang [32] proved that the perceived quality of Internet information would affect peoples’ treatment decisions. Yazdinejad et al. [33] proposed a block-chain-based decentralized verification of patients in a distributed hospital set-up. Ignoring the re-authentication process among distributed affiliated hospitals, the proposed architecture will have a significant impact on network throughput, overhead reduction, response time improvement, and energy consumption. Javed et al. [34] proposed a method called the Cognitive Assessment of Smart Home Resident (CA-SHR) that uses a neuropsychologist to quantify smart home residents’ capacity to perform various tasks on a daily basis using pre-established scoring systems. Shah, Yan, Shah, Shah, and Mamirkulova [10] contributed to the unified signaling theory and Maslow’s hierarchy of needs theory by combining different patient-generated and system-generated signals in order to help patients in deciding on their medical decisions based on their disease risks.

Shah et al. [35] explored the influence of different online signals (online reputation and online effort), offline signals (offline reputation), and disease risk on patients’ physician selection choice for e-consultation during the COVID-19 crisis. The findings suggested that online signals have a significantly positive effect on patients’ e-consultation choice than offline signals.

Although analyzing the quality of online health information is challenging [36], information quality helps to allow patients to obtain better healthcare services, improve the organization’s performance and doctor-patient relationships [37]. To end with this, high information quality leads physicians to adopt a better quality system in healthcare.

### 2.2. Signaling Theory

Spence [38] indicated that signaling mechanism could reduce problems of information asymmetry regarding quality by maintaining equilibrium in which only high-quality seller seeks valuable information to invest in signals. Models of information asymmetry demonstrate that at least one party to a transaction has related information while others do not [39].

It is worth remembering that our research is in the context of the healthcare industry and is unique in the following ways: First, healthcare is a highly information-asymmetric sector. In the case of online healthcare, physicians have more knowledge about their own level of service than the patients. They are also more conscious than patients. Though physicians know their quality of service, patients have little knowledge about this fundamental issue. As a result, physicians will prescribe unnecessary treatments that raise their income even if it may be of little benefit. Because of the limited knowledge, patients do not have a reliable way to assess the quality of the advice they get [40]. This information asymmetry situation produces a power imbalance in transactions, and the transactions often get bugged—a kind of market failure in the worst cases [41].

Second, on PRWs, patients can put pressure on physicians by possibly obtaining a second opinion. In the context of online healthcare, physicians send signals about the quality of service to their patients. Upon receiving this information, patients can change their decision on the quality of service provided by physicians and thus modify their physician choice [40]. Hence, the selection of appropriate signals in the PRWs is vital to the success of these rating websites because different signals can express different types of information and eventually lead to uneven outcomes.

### 2.3. Hypotheses Development

**Figure 1:**
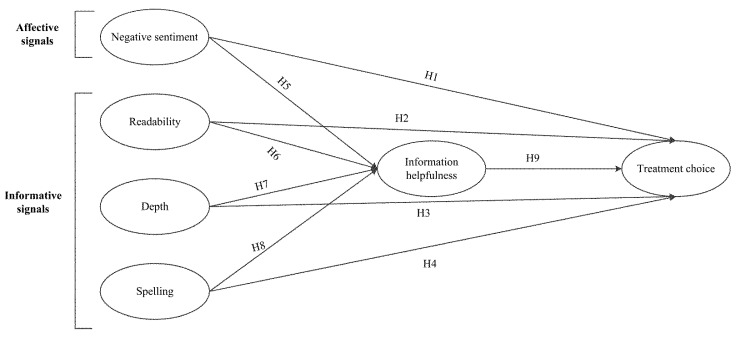
Research model.

Based on the above discussion, this study incorporates five linguistic patterns, which are negative sentiment, readability, depth, spelling, IH, and explore their influence on patients’ choice. Specifically, we categorized negative sentiment as a component of affective signals and readability, depth, and spelling as informative signals. We also examined the mediating effect of IH in the given context. Figure 1 shows the research model.

#### 2.3.1. Affective Signals and Patients’ Treatment Choice

Emotions (e.g., positive vs. negative) significantly influence an individual patient’s ability in his/her treatment decisions [10]. Sentiment refers to the attitude, assumption, or decision stimulated by feelings [42]. User-generated content in PRWs is usually enclosed with sentiment valence (i.e., positive, negative, or neutral sentiment).

In the case of healthcare as a credence good, people may not evaluate the quality of healthcare services even after consumption. Therefore, they seem to favor strongly in reducing losses to boost gains in healthcare decision-making. This implies that those individuals are more prone to perceived damages than benefits when making a healthcare decision in ambiguous or unpredictable circumstances. As healthcare consumers prefer to follow negative OPRs (e.g., low-quality products, monetary losses, etc.) in order to minimize their losses and become more reactive to negative facts, negative OPRs have a greater effect on the patients’ treatment decisions [43]. Given that people detect negative information faster than positive information [42], negative valence PRW–OPRs should have a greater effect on perception creation [44]. Thus, healthcare consumers find negative OPRs more useful than positive OPRs in their treatment decision-making. Following this logic, we demonstrate that negative OPRs provide more predictive, informative, and reliable information regarding the patients’ perceptions of physician service quality than positive OPRs. Thus, we propose that:

**Hypothesis** **1** **(H1).** *The negative sentiment expressed in an OPR on PRW is positively related to patients’ treatment choice.*

#### 2.3.2. Informative Signals and Patients’ Treatment Choice

*Review Readability*: Readability is the degree of comprehension of a piece of text or an article. Online feedback might be comprehensible if these are used as an input variable when making buying decisions [45]. The extant literature has established the readability of information on social media sites as important for consumer acceptance [43,45]. For customer purchase decision-making, a review is reflected to be more useful with sufficient readability than a review that is too lengthy and includes many typographical mistakes, which makes reading challenging for users [16]. Similarly, Yin et al. [46] reported that reading difficulty has a detrimental impact on consumer decision-making. In PRWs, a health information seeker can readily adapt the readability of a review to evaluate the physicians’ performance. Therefore, we hypothesized that the more comprehensible the text, the more helpful the review is considered to be to evaluate the physicians’ performance in terms of patients’ treatment choice.

**Hypothesis** **2** **(H2).** *The readability of an OPR on PRW is positively related to patients’ treatment choice.*

*Review depth*: The concept of review depth refers to the comprehensiveness/elaborateness of the information provided in a review [16]. With the advent of virtual platforms, people post a large amount of online reviews. The overloading of information makes it difficult for us to find the relevant information or ignoring important and critical information [16]. The impact of information overload on the consumer purchase process has been discussed in previous research [43,47]. Short reviews are considered superficial and provide a less detailed evaluation of product characteristics [45]. Long reviews provide more information, including a detailed product overview and its characteristics [48]. An OPR may provide a complete overview of individual health status, medications, and questions or concerns about the physicians’ healthcare quality. Researchers have reported the limits or capacity of purchase decision-makers to process information if the volume is too big or very low. Inadequate information has a detrimental impact on patients’ choice. A comprehensive review may enhance information diagnosticity and reduce the search costs of patients. Keeping all other factors constant, a detailed review not only invokes trust but also provides ample information for patients’ consultation choice [16]. Therefore:

**Hypothesis** **3** **(H3).** *The depth of an OPR on PRW is positively related to* *patients’ treatment choice.*

*Review spelling*: Non-Standard spelling and blank spelling errors are common in user-generated content [44]. The situation may worsen, particularly in the case of PRWs, where users of different backgrounds and different health conditions will impair their ability to spell correctly. Therefore, spelling is another critical factor that leads to obtaining relevant information about physician performance successfully from online peers. In an online social network context, the accurate spelling in one’s review will not only generate a good image of the reviewer’s literacy [49]. Still, it will also effectively communicate the right meaning to the target community. Previous evidence is particularly important for this feature; Ghose and Ipeirotis [50] indicated that the presence of spelling errors in online reviews is negatively associated with the IH. Given the compelling nature of spelling errors in PRWs, we argue that the spelling of reviews on PRWs matters for those individuals who seek informational support to evaluate the physicians’ performance, in turn, leads to patients’ treatment choice. Therefore, we propose:

**Hypothesis** **4** **(H4).** *The degree of correct spelling in an OPR on PRW is positively related to patients’ treatment choice.*

#### 2.3.3. Affective Signals and Information Helpfulness

Another element of a review is its sentiment, which is a brief overall user experience evaluation [47]. Readers can quickly identify the author’s attitude and feeling on the basis of the sentiments. It has been perceived that the more negative the sentiment expressed by the author, the higher will be the IH value [51]. Such negative sentiments can either be exciting or unsatisfactory. For instance, Lee, Jeong, and Lee [42] suggested that negative reviews play a more critical role in consumers’ information processing and decision making. When patients read negative online user feedback about a physician, for example, they may find out that the physicians’ services are of poor quality. This example indicates that negative OPRs for the patients’ consultation decision-making process are more informative and credible; thus, negative OPRs can be viewed as more beneficial than positive OPRs. OPRs with negative sentiment will be more influential. A more compelling review ensures that readers have a better chance of understanding a review. This implies that readers will consider the helpfulness of the review and vote for it. Hence, we propose that:

**Hypothesis** **5** **(H5).** *An OPR expressing more negative sentiment will receive more helpful votes.*

#### 2.3.4. Informative Signals and Information Helpfulness

The main element of a review is the review content. A review should be reliable or easy to understand without potential conflicts to provide information effectively [52]. Readability, judged by its writing style, refers to how easily readers could understand the text. The readability of the text denotes the author’s social status, level of education, and social hierarchy [16]. An OPR with high readability should also be regarded as more accurate than reviews with low readability. If an OPR is accurate or easy to understand, then it would spread its meaning to more people. Thus, a more readable OPR will receive more helpful votes. Therefore:

**Hypothesis** **6** **(H6).** *A more readable OPR will receive more helpful votes.*

The quality of a review can be used as a proxy to determine its helpfulness for consumers in making informed buying decisions. Since a review is a source of information, its usefulness depends on how much information there is in its textual content [53]. In this regard, researchers have shown that the review depth was positively correlated with the perceived helpfulness of a review [48]. A possible justification for this evidence could be that longer reviews may contain more detail than shorter ones [45]. Therefore, healthcare consumers are supposed to consider longer OPRs contained detailed information as being more effective in making their health consultation decisions than shorter ones. Hence, we propose that:

**Hypothesis** **7** **(H7).** *An OPR with detailed information will receive more helpful votes.*

A review with sufficient readability is considered more valuable to users than a review, which is hard to read and contains multiple typographic errors. While many reviewers worry about the accuracy of the spelling when writing online reviews, spelling errors can lead to reading problems. Forman et al. [54] reported that spelling errors have a negative effect on the review helpfulness and readability of a review. Ghose and Ipeirotis [50] found a negative association between online reviews and helpful votes received by the review. In the online healthcare context, we claim that the spelling of an OPR matters for efficient information dissemination. An OPR with the right spelling can efficiently transfer accurate and helpful information to information seekers. Hence, we propose that:

**Hypothesis** **8** **(H8).** *An OPR with correct spelling will receive more helpful votes.*

*Information helpfulness signal*: IH is the component of information quality that reflects the user’s perception regarding the reliability and relevancy of the information. Existing feedback in the form of online reviews on the Internet may be useful or not because usefulness perception leads customers’ intention of information adoption for purchase decisions [23,25].

In a similar vein, IH influences the patients’ consultation choice. From a physician’s perspective, defining the features of an online health information retrieval experience and embedding those features in websites that are perceived to be high quality may lead to customer satisfaction [55,56]. Wu [21] indicated that the perceived usefulness of a system significantly influences the continuance use of online health communities in achieving health-related goals. A system higher in the perceived IH offers a positive user-performance relationship. Scholars also suggest that perceived usefulness/IH/value is the antecedent of consumer behavior [57,58]. For these reasons, we propose that helpful information on a PRW leads to assist users in positively evaluating the quality of physician healthcare services, lead to positive behavior toward that physician.

**Hypothesis** **9** **(H9).** *IH on PRWs is positively related to patients’ treatment choice.*

Further, we argue that patients will make their treatment decisions based on helpful reviews from peer patients. Patients will decide to choose a physician if the information regarding physician service quality is really helpful. Hence, physicians can improve patients’ attitudes by providing useful information to the patients on PRWs. These arguments are consistent with prior findings in the IH literature that link different linguistic signals to review helpfulness and consumer behavior [4,5,21,23]. Hence, we propose that:

**Hypothesis** **10** **(H10).** *IH mediates the relationship between affective signals, information signals, and patients’ treatment choice.*

## 3. Methods

### 3.1. Sample and Data Collection

We test our research hypotheses by collecting data from Healthgrades.com (accessed on 11 August 2020) and Vitals.com (accessed on 11 August 2020), included among the highest traffic ranking websites in the U.S.

In June 2020, we used an automated network spider coded in Python 3.6 to scrape all of the relevant physician profiles from Healthgrades and Vitals. Data were collected covering a period of COVID-19 epidemic outbreak in the U.S from December 2019 to June 2020. So far, this novel coronavirus has caused 2,537,636 confirmed cases with 126,203 mortalities [59]. Clearly, a significant problem during the epidemic was the lack and scarcity of healthcare services. The provision of timely and effective health services during the pandemic period proved to be overwhelming because of inadequate protective equipment, travel restrictions, lockdown, and the possibility of spreading diseases to patients and physicians [60]. Hospitals can increase the effectiveness of their healthcare facilities by replacing physical treatments with virtual technologies in order to reduce and monitor the spread of the pandemic.

For data collection, we chose 10 disease specialties that contain the highest number of active physicians in the U.S [61]. Data were collected from 10 leading U.S states based on the number of active specialist physicians (i.e., California, New York, Texas, Florida, Pennsylvania, Ohio, Illinois, Massachusetts, and New Jersey) [62]. Our final data set include a non-random selection of 5521 physicians with a total of 52,340 reviews from 10 disease specialties.

### 3.2. Measurements

**Figure 2:**
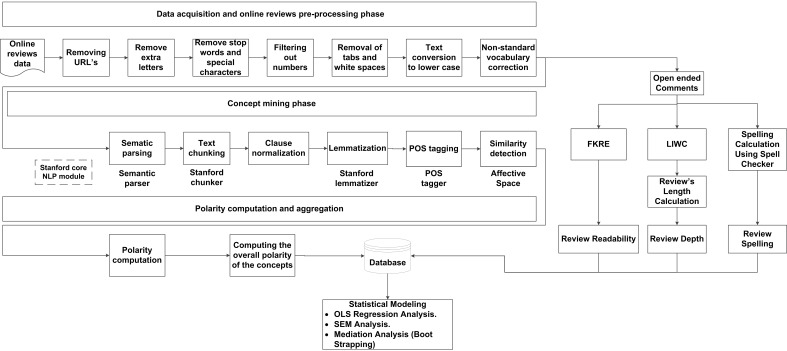
Variable measurement and analysis techniques.

Table 1 shows the variables in the analysis and their definitions. The unit of analysis was the individual OPRs. Figure 2 shows the overall structure of the variable measurement and proposed analysis procedure.

First, we pre-process the raw data in the form of OPRs. Second, we mine different concepts from the pre-processed OPRs using Stanford core NLP modules (parsing, chunking, normalization, lemmatization, POS tagging), and calculate the similarity match between different concepts. Third, we apply the sentiment analysis method to compute the polarity of concepts. Fourth, we use the FKRE to calculate the review readability, LIWC toll to calculate review depth, and spell checker for review spelling checking. Finally, OLS regression analysis, SEM analysis, and boot-strapping-based mediation analysis were performed to determine the impact of different linguistic features on patients’ choice.

#### 3.2.1. Dependent Variable

Patients’ choice is measured by four dimensions. Patients assign the quality ratings for physician services; they received in offline hospitals. These ratings ranged from 1–5 with 1–2 being negative, 3 as neutral, and 4–5 as positive. Hence, quality ratings, the number of blogs that the physician has initiated, the number of physician’s healthcare articles, and the physician number of replies to patients are used to measure patients’ choice. All four dimensions are averaged to obtain a composite variable.

#### 3.2.2. Independent and Mediating Variables

*Negative sentiment*: In order to determine the strength of negative opinion, an emotional response (sentiment score) of users toward a provider is computed. For this purpose, the current study adopted a hybrid approach (sentic computing) that follows the state-of-the-art text mining techniques discussed in previous studies [63,64].

*Review readability*: Flesch–Kincaid Reading Ease (FKRE) is used to measure the readability level of a review (see Equation (1)). FKRE [65] analyses the complexity of the text in order to determine the number of years of education that would be needed for someone to understand the text being assessed.

(1) $$\mathrm{FKRE}\;=\;206.835\;-\;\left(1.015\times \;\frac{\#\;of\;words}{\#\;of\;sentences}\right)\;-\;\left(84.6\;\times \;\;\frac{\#\;of\;syllables}{\#\;of\;words}\right)$$

Score Range = 0–100

=>The score between 60 and 70 is largely considered acceptable.

*Review depth*: Review depth is the number of words in a review. Review depth is calculated using the natural language processing (NLP) tool, that is, linguistic inquiry and word count (LIWC), which is a text-mining program [66].

*Review spelling*: We use an open-source spell checker software (The software Language Tool is available at https://languagetool.org (accessed on 29 September 2020 and 2 October 2020)) to compute the Jaro–Winkler similarity [67] between the original text and the corrected text as the proxy of the PRW review’s spelling. The Jaro–Winkler similarity score ranges between 0 and 1. The higher the similarity between the original and the updated text, the closer the metric reaches 1.

*Information helpfulness* (*IH*) *as a mediator*: On PRWs, there is an accumulative helpfulness vote that results from other reviewers who vote on the effectiveness level of each review. The helpfulness value of the review increases with the increase in the number of helpfulness votes for an OPR. Following the ground truth, we assume that the IH variable here has a continuous value and is measured as the ratio of helpful/useful votes to the total votes. From our dataset, the non-voted reviews were removed to reduce the noise.

#### 3.2.3. Control Variables

We controlled a number of other variables that could theoretically explain patients’ treatment choice. These control variables include: (1) Physician title (*Title*) in offline hospital, (2) practical experience (*Exp.*), and (3) physician gender (*Gender*).

### 3.3. Machine Learning Sentiment Analysis

Choosing a suitable approach for sentiment mining relies on the needs of the analysis. Sentic computing has been used to solve various cognitive-inspired issues, such as classifying natural language text (positive or negative). A hybrid approach to sentic computing and opinion mining incorporates knowledge-based methods and statistical approaches to identify opinions and sentiment calculations from natural language text [18,63].

### 3.4. Pre-Processing of Online Reviews and Concept Mining

**Figure 3:**
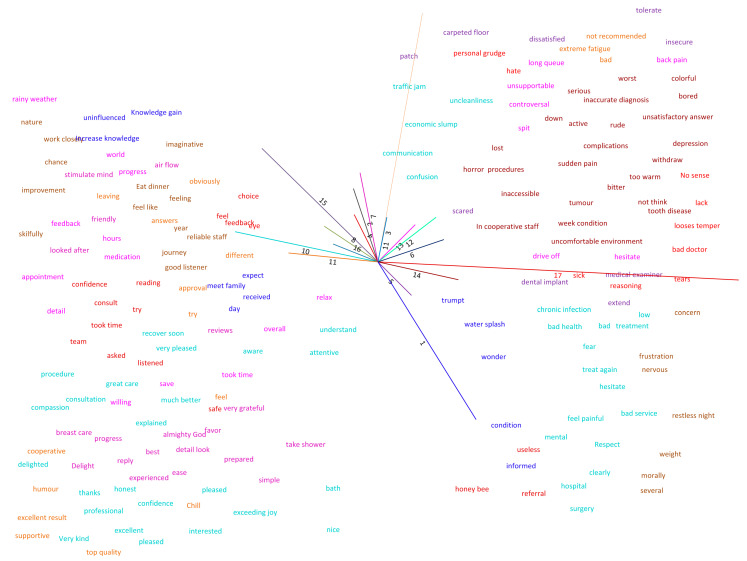
Affective space.

After obtaining OPRs in Section 3.1, the raw data is cleaned using Stanford NLP tools, as shown in Figure 2. To mine the common-sense concepts from the text, the Stanford Chunker [68] is used first to chunk the input text; then, semantic parsing is performed using a semantic parser [69]. The semantic parser breaks sentences into clauses first and then decomposes such clauses into bags of concepts. Concepts are transformed into vector space modeling (VSM) built from WordNet Affect and Concept Net. In VSM, each concept is represented as a point in a vector space with one dimension for a term in the vocabulary [64], as shown in Figure 3. VSM captures the semantic and affective similarities between the concepts and performs analogical reasoning quickly and efficiently [69]. The purpose of concept similarity detection is to compare a given concept with others in the database and thus minimize data sparsity. In addition, to calculate the cross-correlations between concepts, a dimensionality reduction method called truncated singular value decomposition is implemented on the matrix representation of AffectNet.

The polarity score (*p*) of a sentence (*d*) and the overall sentiment score of a review is computed using Plutchik’s wheel on human emotions [70], as shown in Equations (2) and (3), respectively.

(2) $$p\;\;=\;\;\overset{N}{\underset{i=1}{\sum }}\;\frac{Pleasantness\;(c_{i})\;+\;\left|Attention\;(c_{i})\;\right|\;-\;\left|Sensitivity\;(c_{i})\;\right|\;+\;Aptitude\;(c_{i})}{3N}$$

(3) $$Opinion\_score(d)=\frac{\sum_{p_{i}\in d}polarity(p_{i})}{N}$$

where:

*n* = number of concepts

3 = normalization feature

Polarity *p* ranges [−1, +1].

For a given sentence, SenticNet [18] and extreme machine learning [64] determine the opinion score between −1 to +1, where −1 denotes extremely negative, and +1 indicates extremely positive. Iterating the above procedure can result in the opinion score for other reviews in the dataset.

### 3.5. Empirical Model

To model the treatment choice as a function of independent variables and control variables, our empirical model is shown below:

(4) log (Treatment choicei) = β0 + β1 (Scorei) + β2 (Readabilityi) + β3 log(Depthi) + β4 (Spellingi) + β5 log (IHi) + β6 (Titlei) + β7 (Experiencei) + β8 (Genderi) + zi

## 4. Results

**Figure 4:**
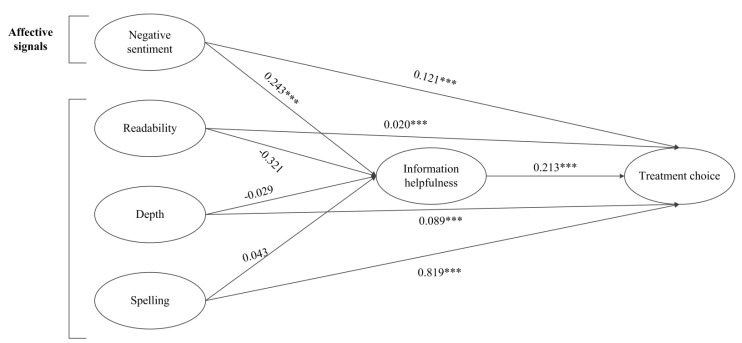
Structural model results. *** *p* < 0.001.

Table 1 and Table 2 present the descriptive statistics and correlations of the variables, respectively. It is interesting to note from Table 2 that for this analysis the VIF values of our independent variables were below 10, hence multicollinearity might be ignored [71]. All our empirical data were analyzed using STATA and AMOS (version 23.0). We ran the ordinary least squares (OLS) regression on our dataset. Testing of the hypotheses was performed by exploring the structural model results using patients’ treatment choice models, which are presented in Table 3 and Figure 4.

### 4.1. Analysis Results for Direct Effects

As shown in Model 1, it can be observed that title (*β* = 0.146, *p* < 0.001) and experience (*β* = 0.241, *p* < 0.01) have positive effects on the treatment choice, while gender (*β* = 0.010, *p* > 0.001) has an insignificant effect onto the treatment choice. From Model 2, it is notable that score (β = 0.121, *p* < 0.001), readability (β = 0.020, *p* < 0.001), depth (β = 0.089, *p* < 0.001), spelling (β = 0.819, *p* < 0.001), and IH (β = 0.213, *p* < 0.001) all have a positive effect onto the treatment choice. Therefore, H1–H5, are all supported. The results found in H1 through H5 generally agree with previous studies demonstrating the positive impact of different features of message on consumer choice [4,16,42,43,44,48,50]. In addition, the results show significant F values and adjusted-R^2^ values, which lie within the defined threshold level [72].

### 4.2. Results for Mediation Analysis

We tested our hypotheses H5–H10 using structural equation modeling (SEM) with the AMOS 23.0. The results are shown in Table 4. The output of SEM indicates that the score (β = 0.243; *p* < 0.001) shows a significant and positive relationship with IH, in line with the previous studies of the influence of emotions on IH [73]. In contrast, insignificant relationships were found between readability (β= −0.312, *p* = insignificant), depth (β= −0.029; *p* < 0.005), spelling (β = 0.043; *p* = insignificant) and IH.

The overall SEM fit provided a value of χ^2^/df = 2.441, which is below the recommended threshold of 3. The CFI = 0.939, NFI = 0.916, TLI = 0.925, root mean square error of approximation (RMSEA) = 0.06 RMSEA; thus, all were above the suggested threshold [71]. Hence, the SEM shows a good fit.

To test for hypotheses H5–H10, the mediating effect of perceived IH was investigated using the bootstrapping method as recommended by Preacher and Hayes [74], and AMOS 23.0. The bootstrapping method is a particularly effective approach to alternative methods such as the Sobel test [75]. In particular, we tested whether score, readability, depth, and spelling have indirect effects on treatment choice through the mediation of perceived IH. We first analyzed the direct effects estimation results without a mediator, the direct results after the mediator (satisfaction) are inputted, and the indirect results. If the indirect effect is significant, then a mediation effect can be established [71]. Table 5 also presents the indirect effects of four independent variables on treatment choice through perceived IH.

First, the indirect effect of the score on treatment choice was positive and significant (95% CI = [0.492, 0.251]). According to Hair, Black, Babin, and Anderson [71], when both the direct and the indirect effects from X to Y are significant, a partial mediation effect occurs; where the direct effect is insignificant when the mediator is introduced, and the indirect effect is significant, the full mediation effect is achieved.

If the direct effect was never significant, but there is an indirect effect, then there is an indirect effect of mediation. In our analysis, direct impact without a mediator and its direct impact with a mediator of the score were both insignificant; hence, this result shows that IH did an indirect mediation role. Second, the indirect impact of readability on treatment choice was not significant (95% CI = [0.035, −0.642]), thus suggesting that IH did not have a mediation effect. Third, the indirect impact of depth on treatment choice was significant (95% CI = [0.211, 0.069]). Since both were significant for its direct effect without a mediator and its direct effect with the mediator, it indicates that IH had a partial mediation effect. Finally, the indirect effect of spelling on IH was insignificant (95% CI = [0.089, −0.007]), indicating that IH did not have a mediation effect. These results confirm potential mediating impacts of IH raised by previous research [4].

## 5. Discussion

### 5.1. Discussion of the Results

Linguistic signaling is an interesting and complex topic, particularly in an online environment where there are often substantial asymmetries to information. Investigating the impact of different linguistic signals on patients’ treatment choice during the COVID-19 epidemic period can be a major challenge. Nevertheless, we still need to see a theoretical framework considering linguistic signaling within PRWs. A comprehensive understanding of linguistic signaling in the online healthcare environment provides a theoretical foundation for patients’ choice toward a specific physician.

First, H1 is supported, which means that the positive relation between negative sentiment and patients’ health consultation choice has been verified. Previous studies have proven that negative emotions positively influence user behavior [49,51]. In our context, when users read negative opinions about healthcare providers, they will decide whether to consult a particular physician or not.

Second, H2, H3, and H4 are supported significantly, which means that informative signals positively influence users’ decision-making process. These results are consistent with extant studies [46,49,76]. Informative signals include review readability, depth, and spelling. When users read online reviews and find a review to be more readable, comprehensive, and accurate about a particular provider, they will trust in this online review to consult a particular physician.

Third, H5 is significantly supported, that is to say, patients’ negative sentiments about physician’s service quality have significant effects on perceived IH. The positive relation between user sentiments and IH has been tested before [48,76]. The results of the present study showed that negative emotions in reviews showed a positive effect on review helpfulness. This finding can be considered in light of various prior investigations. When a service fails, customers may vent their frustrations in online reviews to let others know about their bad experience [42,77]. Potential customers who read reviews may appear to place a higher value on losses than on profits, based on their perceptions [49]. This argument is in line with the findings of Cacioppo and Berntson [78], who found that negative input, has a stronger impact on behavioral expressions than positive input.

Fourth, H6, H7, and H8 are not supported, which means that readability, depth, and spelling are not supported toward the IH. These results are not in line with the previous findings [16,44,79]. The probable explanation behind these results is that when consumers search information online regarding the provider for their health consultation, they do not perceive the readability, depth, and spellings of online reviews to be helpful; thus contradicting the earlier findings. However, regarding H9, our study findings showed that IH is significantly and positively related to treatment choice, in line with previous findings [4,80,81]. Patient decision-making can be considered as perfect if a large amount of electronic word of mouth activity exists and these reviews are helpful at the same time, which indicates the physician is popular and attracts patients’ attention more than the other physicians.

Finally, regarding H10 the current study explores the mediation effect of IH between affective and informative signals and patients’ health consultation choice. In our context, the PRWs aim to provide users with more helpful or useful information regarding disease consulting services and providers. If users experience high quality of doctor’s service and health information, they will think it more useful to consult a particular physician [82].

### 5.2. Theoretical Contributions

This work adds to theoretical knowledge in a number of ways:

First, we present signaling theory as a relevant theoretical underpinning for our proposed linguistic signals-based paradigm for holistically addressing patients’ information demands. In specific, we examined the affective signals in terms of the negative sentiment and informative signals as the readability, depth, and spelling of online reviews. Prior research in various domains has mainly focused on the effects of one or two signals in isolation [4,73,83]; all signals are not systematically studied in one study, and hence we cannot compare these effects directly due to varied research settings and isolated study outcomes. Therefore, we incorporate work from well-grounded quantitative research and also includes qualitative analysis. Mining review text posted during the novel coronavirus period, the current study helps to bridge the gap from both content and linguistic aspects to the emerging research body. Since limited studies have examined review content focusing on cognitive perceptions (e.g., readability, depth), this study is one of the first to examine precisely how individuals’ emotions implicitly serve as signals for readers to assess patients’ choice toward a physician. When we compare our proposed framework to other physician recommendation systems, we find that ours can assist patients in making better physician selections.

Furthermore, this is the first study to examine the influence mechanism of patients’ treatment decision-making process. On the foundation of different features of messages in the form of OPRs, the OLS regression model, SEM analysis, and mediation analysis are used to analyze the influencing message features on patients’ decision-making. We found that the factors which influence patients’ health consultation choice include negative sentiment, readability, depth, spelling, and IH. Based on the results from the mixed methods, we incorporate those key elements into the patients’ choice model for identifying high-quality physicians. Since we introduced the concept of IH in online healthcare services, we also proposed and tested for the first time a new mediator, IH [4], which explains the effect of linguistic signals on patients’ choice toward physician services. The results showed that the IH mediates the relationships between negative sentiment, review depth, and patients’ treatment choice.

Finally, our information technology-based linguistic signals transmission method is a big data technology, which is more than just an internet technology. It contributes to the theoretical literature on the use of information technology and signaling theory by providing new insights. Moreover, our study is a theoretical extension of information processing, allowing patients and healthcare organizations to exchange information more easily, and enhance information sharing among online healthcare platforms. Furthermore, it expands the application of signaling theory and responds to calls for theory development in healthcare management.

### 5.3. Practical Implications

The study’s findings indicate that technologies such as big data, machine learning, regression analysis, and multi-method analysis can be integrated to benefit the health domain. The findings have important managerial implications for patients and healthcare practitioners, and firms that provide similar high involvement services can benefit from this technology.

First, identifying high-quality providers with the use of internet-based health information technology brings tremendous benefit to patients. The proposed linguistic signals model can help in the improvement of patients’ healthcare service experiences by allowing them to select the most appropriate physicians; hence minimizing patient dissatisfaction caused by sub-optimal medical professionals. Thus, the dispute between physicians and patients may be reduced. Furthermore, the proposed information technology paradigm has the potential to save patients search costs, time, and money.

Second, using information technology to find good doctors may benefit healthcare administrators. There is a significant gap between the vast amount of online healthcare information and the information requirements of users. To bridge this gap, we provide a physician selection choice model for patients that healthcare platforms can utilize to select the best physicians, to increase the user experience, and to increase their website’s efficiency. In practice, this study also provides web designers with an overview of the features of the most valuable knowledge that could help them in designing signals for patients and online healthcare industry reviewers to use during COVID-19 pneumonia [84]. Web designers and moderators can actively examine the linguistic characteristics that invoke useful responses toward providers. In particular, most reviewers use a similar writing style that first helps to communicate information effectively and includes opinions and emotional content. In addition, where certain styles are normative, moderators can educate new users about the language features that the majority prefer (e.g., emotion, length, style, etc.), and/or provide ideas in real-time as reviews are posted, such as different wordings or styles that more closely fit reviews in the PRWs previously. Designers can even take into account an innovative characteristic that can analyze the review content (e.g., length, spelling errors, etc.) to provide an opportunity for evaluating physicians’ performance. Through providing information on crowd assessments and crowd behavior, web designers can make the assessment of healthcare services simpler for customers, thus encouraging buying decisions.

Lastly, this study makes vital contributions to the medical and healthcare industries; the effective and efficient use of negative feedback is of utmost importance for physicians during the COVID-19 pandemic. As patients find negative reviews to be more influential in their treatment decisions than positive reviews, physicians should take decisive steps to resolve the depressed feelings of patients before they become more disenchanted. Physicians may improve review management practices by classifying and/or ranking feedback from the patients’ eyes through their value or helpfulness; for example, usually, first favorable reviews, and then critical reviews with strong emotional statements. Similar to the service quality and recovery paradigm in other domains suggests [85] that patients think positively about a physician after their mistakes have been corrected, compared to how they would look at the physician if high-quality care were delivered. Further, physicians should be vigilant to negative feedback with intense emotional expressions. Such kind of feedback would influence the review site’s profile, which could possibly cause clients to leave for other sites. To maintain strong ties with patients in the digital world, it would be important for the review sites to have clear guidance on posting reviews and online protocols.

### 5.4. Limitations and Future Research

This research has certain limitations that could be addressed in future works. First, we only examined the linguistic features and their impact on consumers’ choice. There are some other characteristics (i.e., review valance and review volume), which may also affect consumers’ choice. Future research should consider these characteristics and their impact on consumers’ behavior. Second, in this study, we collected data for 6 months during the epidemic period; however, research must be carried out over a substantially longer period. As part of our further research, we will continue collecting data at different time points when the epidemic comes to an end.

## 6. Conclusions

With the implementation of digital technology in healthcare, the utilization of PRWs becomes increasingly popular during the COVID-19 epidemic crisis. An OPR that evaluates a physician’s competence would inevitably impact the patients’ choice of a physician on PRWs. This research examined the impact of affective signals and informative signals on patients’ physician selection. Further, the mediation effect of IH was investigated in the relationship between the linguistic signals and patients’ treatment choice. Our findings revealed that negative sentiment, review readability, review depth, review spellings, and IH have positive effects on patients’ treatment choice. Moreover, the IH mediates the relationship between negative sentiment, review depth, and treatment choice. Theoretically, this paper establishes a research model based on signaling theory to understand the linguistic signaling mechanism on patients’ behaviors and adds significantly to the literature concerning PRWs. In practice, our research findings suggest that web designers and physicians better deal with the impact of OPRs on them.

## Figures and Tables

**Table 1:** Variables definition.

Variables	Definition	Analytical Method	Mean	Std.	Min.	Max.
**Dependent Variable** Treatment choice	Rating—Physician quality ratings (Negative = 1–2, Neutral = 3, Positive = 4–5) Blogs—The number of blogs initiated by a physician (logarithmic value) Articles—The number of articles published by a physician (logarithmic value) Replies—The number of replies to patients by physician		4.23 3.35 0.14 5.3	0.55 20.4 10.05 112.6	1 0 0 0	5 25 45 48
**Independent Variables** Negative sentiment	Score—Sentiment score of a review (in the range [−1, +1], where −1 is the strongest negative opinion)	Sentiment analysis	−0.34	1.22	−1	+1
Review readability	Readability—The ease of reading score of a review	FKRE	0.84	0.22	–	–
Review depth	Depth—The number of words in the review	LIWC	67.13	156.13	–	–
Review spelling	Spelling—The level of spelling of the review (posted version vs. corrected version)	Spell checker software	98.15	112.12	–	–
**Mediating Variable** Information helpfulness	IH—Ratio of helpful/useful votes to the total votes		0.92	0.07	0	1
**Control Variables** Physician title	Title—Physician title in offline hospital “1” if medical doctor, “0” otherwise		0.91	0.51	0	1
Practical experience	Experience—Practical experience refers to how long a physician has provided professional service. Practical experience was coded with “0” for 0–10 years experience, “1” for 11–20 years experience, and “2” for more than 20 years experience		1.34	0.43	0	2
Physician gender	Gender—Gender was coded with “0” for male and “1” for female		0.89	0.49	0	1

**Table 2:** Variables correlations.

Variables	1	2	3	4	5	6	7	8	9	10
1. Treatment choice	**1.00**									
2. Score	0.25	**1.00**								
3. Readability	0.01	0.02	**1.00**							
4. Depth	0.12	0.15	0.21	**1.00**						
5. Spelling	−0.03	−0.02	−0.04	−0.05	**1.00**					
6. IH	0.32	0.41	0.25	0.21	0.23	**1.00**				
7. Title	0.19	0.24	0.19	0.08	0.12	0.18	**1.00**			
8. Experience	0.32	0.28	0.32	0.02	0.12	0.13	0.10	**1.00**		
9. Gender	0.12	0.14	0.21	0.23	0.20	0.29	0.32	0.28	**1.00**	
10. Title	0.13	0.151	0.114	0.21	0.19	0.22	0.24	0.25	0.23	**1.00**

**Table 3:** Estimation results.

Variables	Model 1	Model 2
Constant	0.121 (0.011)	0.111 (0.015)
Title	0.146 *** (0.004)	0.041 *** (0.003)
Experience	0.241 ** (0.028)	0.261 ** (0.048)
Gender	0.010 (0.015)	0.016 (0.027)
Score		0.121 *** (0.004)
Readability		0.020 *** (0.001)
Depth		0.089 *** (0.007)
Spelling		0.819 *** (0.145)
Log (IH)		0.213 *** (0.012)
Adjusted-R^2^	0.208	0.217
Log-likelihood ratio	429.631	419.765
F	76.683 ***	7.174 ***
*n*	52, 340	52, 340

Note: Standard errors are in parentheses. ** *p* < 0.01; *** *p* < 0.001.

**Table 4:** Structural equation modeling and bootstrapped mediation analysis results.

Model’s Goodness of Fit		Hypotheses	Relationship	Β	T	
χ^2^/df	2.441	H5	Sentiment → IH	0.243 ***	4.021	Supported
NFI	0.916	H6	Readability → IH	−0.312	−1.432	Not supported
TLI	0.925	H7	Depth → IH	−0.029	−0.543	Not supported
CFI	0.939	H8	Spelling → IH	0.043	1.243	Not supported
RMSEA	0.051					

Note: *** *p* < 0.001.

**Table 5:** Bootstrapped mediation analysis model.

			Indirect Effect CI at 95%		
Hypothesis	Direct Effect without Mediator	Direct Effect with Mediator	Upper Bounds	Lower Bounds	Mediation Category
Score → IH → treatment choice	−0.013	−0.034	0.492	0.251	Indirect mediation
Readability → IH → treatment choice	−0.040	−0.034	0.035	−0.642	Insignificant
Depth → IH → treatment choice	0.197 *	0.156 *	0.211	0.069	Partial mediation
Spelling → IH → treatment choice	0.265 *	0.203 *	0.089	−0.007	Insignificant

Note: * *p* < 0.05.
